# Organic Solid-State Electrolyte Synaptic Transistors with Photoinduced Thiol–Ene Cross-linked Polymer Electrolytes for Deep Neural Networks

**DOI:** 10.1021/acsmaterialslett.4c02511

**Published:** 2025-01-23

**Authors:** Qun-Gao Chen, Wei-Ting Liao, Rou-Yi Li, Ignacio Sanjuán, Ning-Cian Hsiao, Chan-Tat Ng, Ting-Ting Chang, Antonio Guerrero, Chu-Chen Chueh, Wen-Ya Lee

**Affiliations:** † Department of Chemical Engineering and Biotechnology, National Taipei University of Technology, Taipei 106344, Taiwan; ‡ Institute of Advanced Materials (INAM), Universitat Jaume I, 12006 Castelló, Spain; § Department of Psychology, National Chengchi University, Taipei 11605, Taiwan; ∥ Research Center for Mind, Brain & Learning, National Chengchi University, Taipei 11605, Taiwan; ⊥ Department of Chemical Engineering, National Taiwan University, Taipei 10617, Taiwan

## Abstract

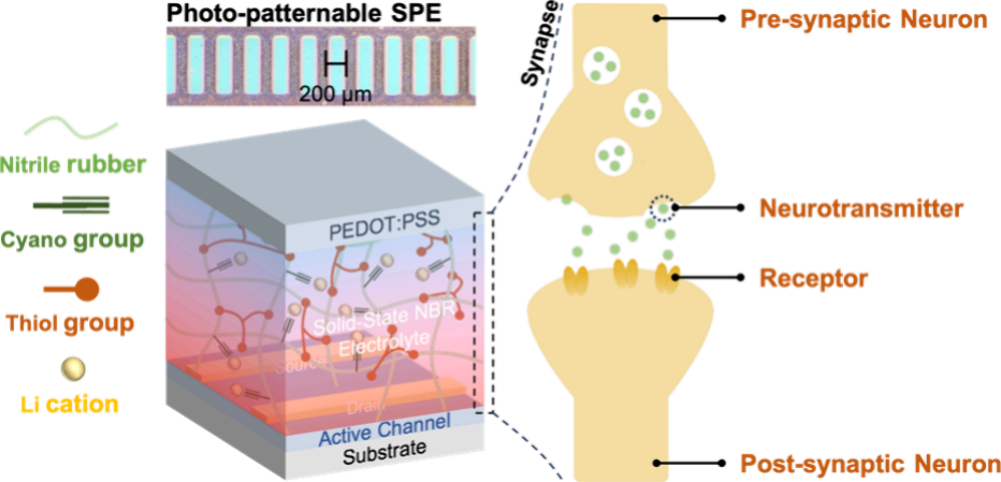

In this work, we describe a solid-state polymer electrolyte (SPE)-based electrolyte-gated organic field-effect transistors (EGOFETs) consisting of a thiol–ene-assisted photo-cross-linked nitrile butadiene rubber (NBR) network embedded with lithium bis(trifluoromethanesulfonyl)imide (LiTFSI) electrolyte. The photocurable SPE film can be patterned with different dimensions by photolithography and exhibits excellent electronic properties and crucial synaptic behavior. The photocurable NBR/LiTFSI EGOFET exhibits a high transconductance of 11.9 mS and a high on/off ratio of 10^5^ at a scan rate of 40 mV/s. Due to the strongly polarized nature of the photo-cross-linked NBR network and Li-ion diffusion, the NBR/LiTFSI device exhibits a significant current hysteresis, enabling synaptic-like learning and memory behavior. The NBR/LiTFSI device demonstrates a DNN of 91.9% handwritten digit recognition accuracy. This work demonstrates the potential of the solid-state NBR/LiTFSI EGOFET in creating highly efficient and low-energy neuromorphic devices.

Synaptic transistors are known for their neuron-like operation, featuring high performance and low energy consumption. To promote the development of neuromorphic chips to meet the demands of artificial intelligence (AI) models and cloud computing. Recently, to overcome the substantial energy consumption and processing time of traditional von Neumann architectures, researchers have pulled inspiration from the human brain,^1,2^ recognizing the immense potential of integrating central processing units (CPUs) with memories in organic field-effect transistors (OFETs) to develop artificial synaptic devices.^3−7^ The gate electrode is regarded as the presynapse, while the drain/source electrodes represent the postsynapse. The gate terminal modulates the channel conductance between the drain and source and mimics the potential changes during the release and reception of neurotransmitters.^8−12^ Furthermore, the synaptic behavior exhibited by OFETs has been employed as adjustable weights in neural networks (NNs) to perform classification tasks, which analyze the potential of these synaptic transistors for neuromorphic applications.^9,13^

Various OFET-based synaptic devices have been developed to mimic neurons, including ferroelectric transistors,^14,15^ floating-gate transistors,^3^ polymer electret,^16^ ambipolar OFETs,^11,17,18^ and ionic doping OFETs.^19−23^ Among them, organic electrochemical transistors (OECTs),^24−26^ and electrolyte-gated organic field-effect transistors (EGOFETs),^27,28^ which involve electrochemical effects, have attracted much attention. The high capacitance of electrolytes reduces driving voltages,^29−33^ and these devices simulate synaptic characteristics via ion diffusion-induced potential changes. Moreover, ionogels and liquid-state electrolytes contain amounts of organic solvents, raising concerns about long-term stability, mechanical robustness, and safety for high-efficiency chips.^34−36^ In contrast, EGOFETs based on solid-state polymer electrolytes (SPEs) offer superior stability and promise for neuromorphic chips. Previous reports have used common ion-conducting polymers such as PVDF-TrFE and PEO as solid-state polymer electrolytes. However, these SPEs are susceptible to organic solvent degradation, making these polymers challenging to use in large-scale photolithography processes.

This work demonstrates a novel photocurable SPE for EGOFETs (Figure S1).The SPE is synthesized via thiol–ene click chemistry, utilizing nitrile butadiene rubber (NBR) and lithium bis(trifluoromethanesulfonyl)imide (LiTFSI) as the electrolyte matrix. Thiol groups (−SH) from trimethylolpropane tris(3-mercaptopropionate) (TMPMP) react with alkene groups (C=C) in NBR to produce photocurable SPEs. Performance is compared with conventional poly(vinylidene fluoride-*co*-hexafluoropropylene) (PVDF-HFP)/poly(ethylene oxide) (PEO)-based SPEs. The novel photocurable NBR/LiTFSI SPE presented here offers a significant advancement in organic synaptic transistors, enabling the realization of more complex circuits and efficient neuromorphic computing systems.

As illustrated in Figure 1a, the alkene groups in the NBR chain can react with the thiol groups via a thiol–ene radical cross-linking mechanism. Thus, the thiol groups in TMPMP react with the alkene group in NBR to form a robust cross-linked polymer network.^37^Figure 1b shows the characteristics of the cyano group at 2230 cm^–1^. In non-cross-linked NBR, the alkene group has absorption characteristics at 1580 and 1540 cm^–1^. Due to the cross-linking reaction between the thiol group and the alkene group in NBR, these features are weakened after photocuring. This clearly indicates that we have successfully prepared a cross-linked polymer network by photocurable NBR using a thiol-based cross-linker.^38^ To further demonstrate the potential of NBR-based SPEs for large-area manufacturing, we applied the NBR-based SPE film to the photopatterning process through a mask. Figure 1c exhibits two NBR-based SPE patterns with different sizes (550 and 200 μm) produced by photolithography. The overall pattern is clearly defined, demonstrating the excellent patterning capability of the NBR-based SPEs. In addition, we examined the cross-sectional profile of the NBR-based SPEs with a size of 200 μm, as shown in Figure 1d, the thickness of the patterned SPEs is ∼400 nm. Collectively, these results show that our developed NBR-based SPEs can be effectively patterned by the photolithography processes, making it suitable for mass-producing device arrays.

**Figure 1:**
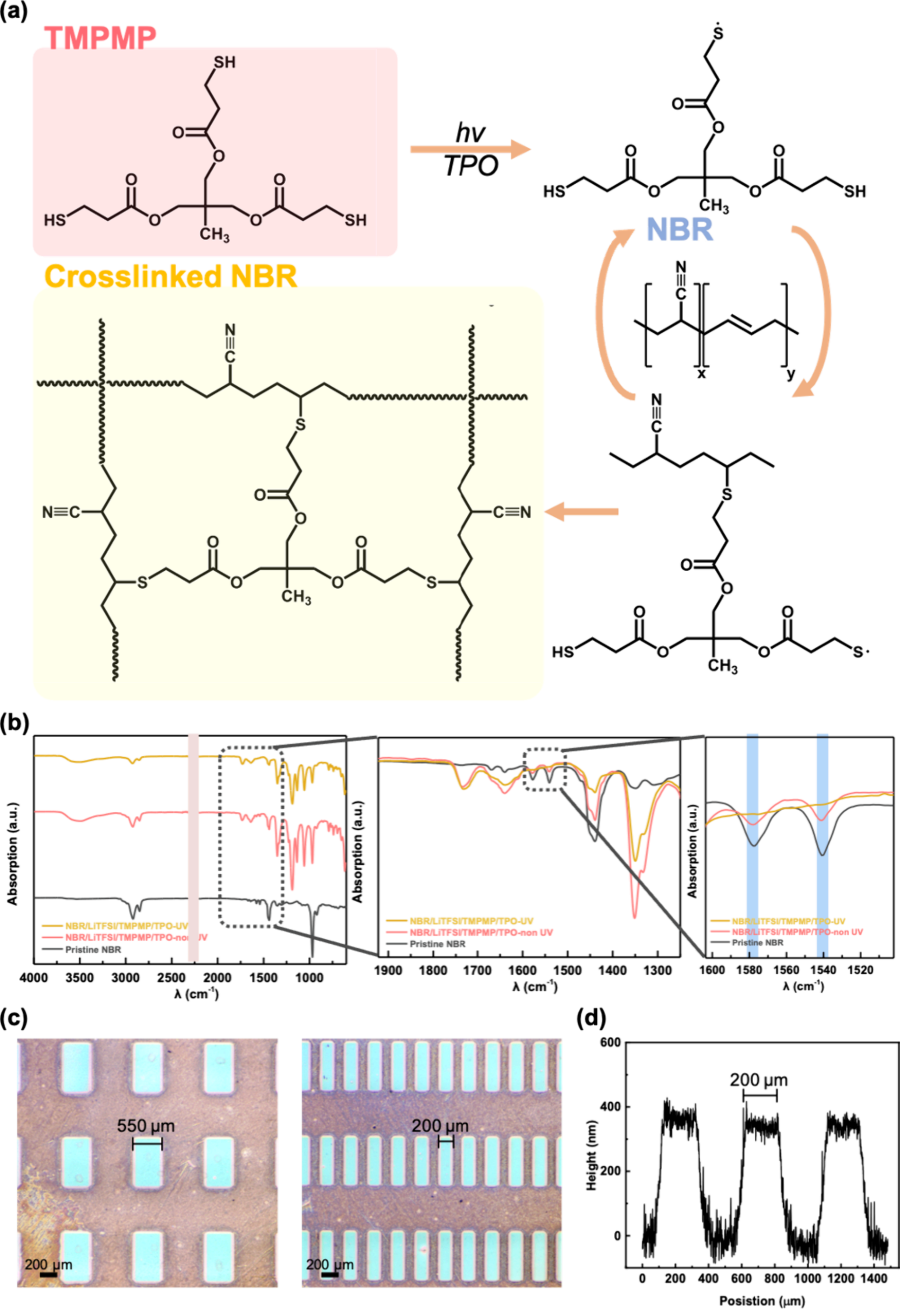
(a) Schematic representation of the thiol-ene radical cross-linking reaction. (b) FTIR analysis of the pristine NBR film and NBR-based SPEs with and without cross-linking. (c) Optical microscope images of patterns with 550 and 200 μm dimensions (scale bar = 200 μm). (d) Cross-sectional profile of the film with a pattern size of 200 μm.

We further analyzed the capacitance of cross-linked NBR at different LiTFSI concentrations (Figure S2a). Comparisons were also made with common SPEs such as PVDF-HFP and PEO (Figures S2b and S2c). All SPEs exhibit a dependence on LiTFSI concentrations, and capacitance increases with the Li^+^ concentration due to ionic polarization caused by ionic doping. Moreover, we explore Nyquist plots of SPEs based on cross-linked NBR, PVDF-HFP, and PEO with LiTFSI (Figure S3a). In the complex impedance plot, a high-frequency was observed in cross-linked NBR, as well as the transmission line of these SPEs. Then, the capacitance frequency plot (Figure S3b) shows the activated ions transport for the PEO-based SPE with an onset for the increase in capacitance from very high frequencies with double-layer capacitance values from 100 Hz. The slope of the capacitance increases relates to the ionic diffusion of the system. The PEO-based SPE enhanced ion migration due to the interaction of Li^+^ with O atoms in the PEO chain.^39,40^ Alternatively, the PVDF-HFP-based SPE showed a constant increase in capacitance with a small slope and a low overall double capacitance value (1 × 10^–6^ F/cm^–2^) even at lower frequencies. This is because the PVDF-HFP-based SPE showed wettability issues that led to impeded ion migration.^41^ Finally, NBR-based SPEs exhibited the high frequency are with a resistance of 3 kΩ observed as a plateau in the capacitance–frequency plots up to frequency values of 100 Hz. We believe that this high resistance arc relates to the presence of the cross-linked matrix that, in turn, will lead to the nonvolatile response of the device. Once, the ions begin to migrate at lower frequencies, it is possible to obtain high double-layer capacitance values, but the onset in ion migration is retarded due to the presence of the high-frequency arc. We summarized the ionic conductivities of cross-linked NBR, PVDF-HFP, and PEO with 25 wt % LiTFSI, which were 1.95 × 10^–7^, 1.22 × 10^–7^, and 3.11 × 10^–5^ S/cm, respectively (Table S1). However, the non-cross-linked PEO-based SPEs lack stability under the influence of electric fields.^42,43^ In comparison, NBR-based SPEs can provide better stability through the cross-linked polymer network formed by photocuring.

Furthermore, we explore the correlation between polymers containing polar functional groups and ions. We calculated the molecular electrostatic potential (MEP) and dipole moments of NBR, PVDF-HFP, and PEO using density functional theory (DFT) at B3LYP/6-31G. Figure 2a shows that the bond lengths of NBR, PVDF-HFP, and PEO are 1.16, 1.41, and 1.46 Å, respectively. The electrostatic potential (ESP) displays a sternly negative region with a functional group polarization. Then, the bond lengths of polar groups in NBR, PVDF-HFP, and PEO increase to 1.17, 1.46, and 1.47 Å, respectively. The results indicate that the interaction of the polar regions with the Li^+^ ion weakens the original C–N, C–F, and C–O bonds. This suggests that the addition of LiTFSI enhances the interaction force between the polar groups and the Li^+^ ion. Therefore, in order to respond to the electric field, Li^+^ ions need to overcome the interaction with the polar functional groups to diffuse to the interface. This results in a decrease in the response speed of Li^+^ ions, which gives rise to significant synaptic properties. Next, we investigated the electrical characteristics of EGOFETs using NBR-based SPEs, where PDBT-*co*-TT was used as a *p*-type channel. Figure 2b shows the transfer curves at a low driven voltage of −1 V, where the clockwise current hysteresis is clearly observed. The higher reverse-scanning current should be attributed to the diffusion of Li^+^ ions in the cross-linked NBR polymer matrix. The interactions between the cyano groups of NBR and Li^+^ ions promote ion transport in the cross-linked SPE.^44^ In the cross-linked NBR doped with 5 wt % LiTFSI, we observed an on/off current ratio (*I*_on_/*I*_off_) of ∼10^5^, but due to the low ionic doping concentration, the transconductance (*g*_m_) is only 0.092 mS (Figure S4). Notably, the cross-linked NBR with 25 wt % LiTFSI exhibited an *I*_on_/*I*_off_ close to 10^6^ and a *g*_m_ of 2.46 mS. The performance of the overionic doped LiTFSI (45 wt %) device is lower than expected, which is related to its surface roughness. In the next section, we will discuss the dependence of ionic doping concentration and film roughness on SPEs. For comparison, we also studied the electrical performance of EGOFETs using PVDF-HFP- and PEO-based SPEs. The typical clockwise current hysteresis was also observed in the PVDF-HFP- and PEO-based EGOFETs. (Figures S5a and S5b**)**. We clearly defined the linear region of these EGOFETs (Figure S6). The capacitance of the SPEs based on NBR, PVVDF-HFP, and PEO increases with increasing LiTFSI content (Figure S4). This is due to the high ionic doping in the polymer matrix, which induces more change generation through the electric field. Therefore, the LiTFSI content in the SPEs can be controlled to achieve the best insulation properties and ionic conductivity. It should be noted that *I*_on_/*I*_off_ ratios of the cross-linked NBR-, PVDF-HFP-, and PEO-based EGOFETs differ significantly, with values of 10^5^, 10^2^, and 10^2^, respectively. The cross-linked NBR-based EGOFETs shows the highest *I*_on_/*I*_off_ ratio, which is attributed to the presence of the cross-linked NBR network. Compared to PVDF-HFP and PEO, the cross-linked NBR is more susceptible to external gate electric field, as the dipole polarization of NBR alters the carrier distribution at the channel/SPE interface and reduces the device off-current.^45^ Then, we investigated the performance of the cross-linked NBR-based EGOFET at different scan rates. As shown in Figure 2c, the device exhibits a rate dependence related to *g*_m_ of 11.9, 2.30, and 0.79 mS, corresponding to scan rates of 40, 80, and 120 mV/s, respectively. The *I*_on_/*I*_off_ ratios at these scan rates decreased from 10^5^ to 10^4^. At low scan rates, the Li^+^ ions and cyano groups in the cross-linked NBR-based SPE can respond fully and accumulate more charges, thereby generating more changes in the active channel and producing a higher current output. In contrast, the SPEs do not have enough response time, resulting in a two-order-of-magnitude difference in *g*_m_ under a fast scan rate. It is worth noting that the cross-linked NBR-based EGOFET has a high *g*_m_ of 11.9 mS, which is significantly higher than the PVDF-HFP-based EGOFET (0.42 mS), and the PEO-based EGOFET (1.12 mS). Overall, the cross-linked NBR-based SPE enables the derived EGOFET to exhibit excellent electrical performance with low-driving voltages, high *g*_m_, and a high *I*_on_/*I*_off_ ratio.

**Figure 2:**
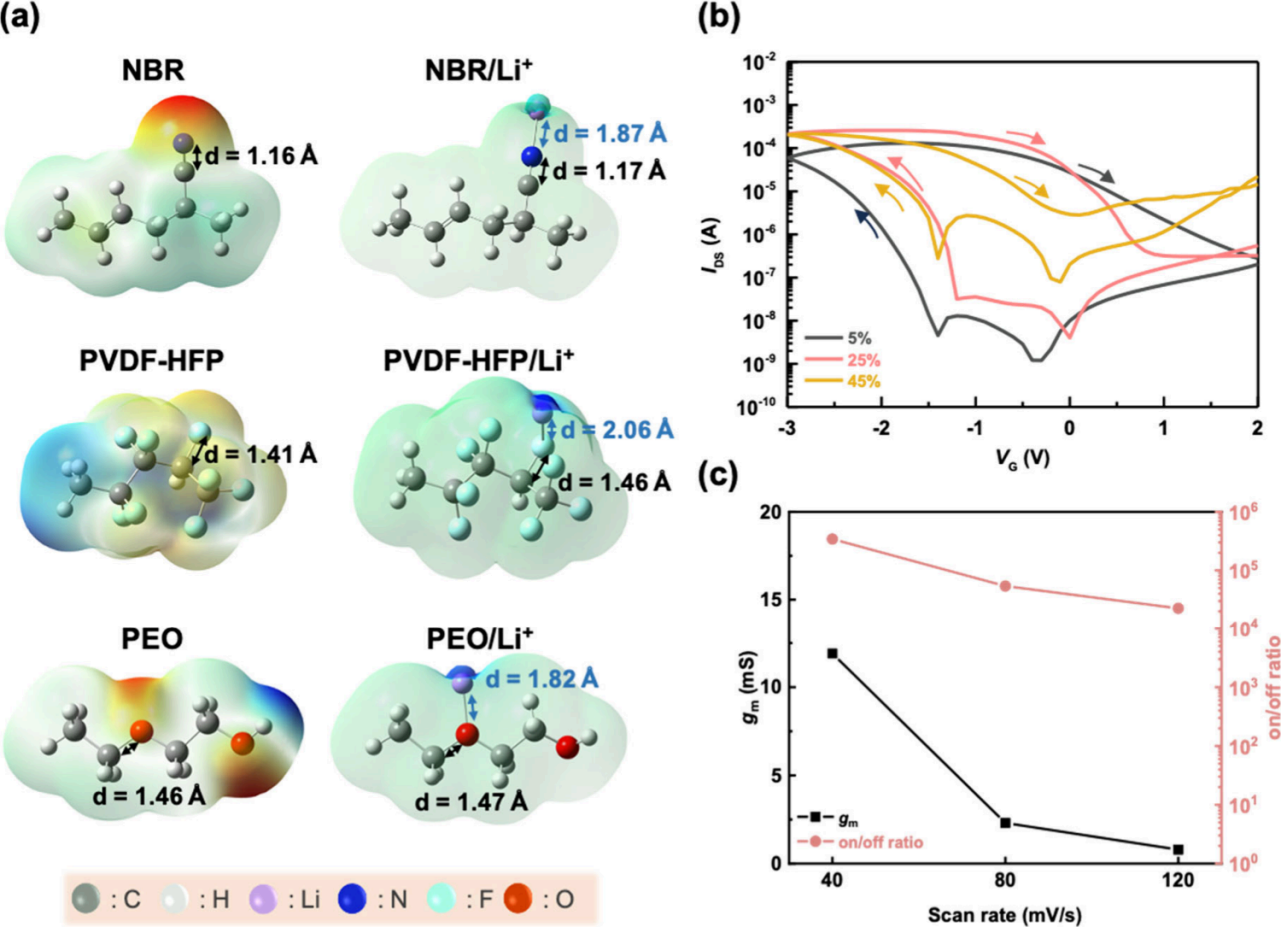
(a) MEP mappings before and after the addition of Li^+^ ion for non-cross-linked NBR, PVDF-HFP, and PEO. (b) Transfer curves of PDBT-*co*-TT EGOFET based on the cross-linked NBR-based SPEs with different LiTFSI contents (5, 25, 45 wt %). (c) Scanning rate-dependent transfer curves of PDBT-*co*-TT EGOFET based on the cross-linked NBR/LiTFSI (25 wt %) SPE.

We noticed that the use of UV curing to prepare the cross-linked NBR-based SPEs caused different properties, which, in turn, affected the performance of the device when preparing the EGOFET (Figure S7a). We use the cross-linked NBR-based SPE attachment surface to define the front and reverse sides of the film (Figure S7b). Although both configurations achieved a high *I*_on_/*I*_off_ = 10^5^, the EGOFET with the front-side cross-linked NBR-based SPE displays a very low *g*_m_ of only 0.24 mS. Notably, the device with the reverse-side cross-linked NBR-based SPE shows a higher *g*_m_ of 1.85 mS under the same operating conditions. These configuration differences clearly reveal the critical role of the channel/SPE interface in EGOFETs. To further investigate this effect, we studied the morphology of both sides of the cross-linked NBR-based SPEs. First, we analyzed the surface morphology of the reverse-side of the cross-linked NBR-based SPE. The root-mean-square surface roughness (*R*_rms_) of the reverse-side surface of the NBR-based SPEs (Figures S8a–S8c) changes with LiTFSI content, which is 0.93, 0.42, and 1.89 nm for 5, 25, and 45 wt %, respectively. Meanwhile, the front-side surface of the NBR-based SPEs (Figure S8d–S8f) also shows a similar trend. The surface roughness is 2.58, 1.23, and 3.63 nm, corresponding to 5, 25, and 45 wt %, respectively. Connected with the high roughness at high LiTFSI content, different aggregates, such as scales or fibers, are formed for the front and reverse side of the films, respectively. Figure 3d summarizes the surface roughness of the front and reverse sides of the cross-linked NBR-based SPEs. It is worth noting that too little or too much LiTFSI content can lead to surface aggregation, as shown in Figures 3a and 3c. Figure 3b shows that the appropriate LiTFSI concentration (25 wt %) results in the smoothest surface, enabling the derived EGOFETs to exhibit the highest electrical performance. There are two possible reasons for the difference in surface roughness between the front and reverse sides of the cross-linked NBR-based SPE: (a) uneven distribution of LiTFSI phase separation occurred during the preparation of the SPE film, resulting in uneven distribution of LiTFSI in the NBR; (b) different cross-linked density: during the UV curing process, the cross-linked density on the front-side of the SPE is higher than the reverse side of the SPE. Therefore, we further used time-of-flight secondary ion mass spectrometry (TOF-SIMS) to analyze the changes in the elemental distribution in the cross-linked NBR/LiTFSI (25 wt %) SPE to clarify the exact reason. Note that the key elements in NBR are C^+^ and N^+^ while LiTFSI has Li^+^, F^+^, S^+^, N^+^, and O^+^. As shown in Figure 3e, the substrate contributes to the intensity of Si^+^ and O^+^, which gradually increases and stabilizes at 4 μm. The other elements are evenly dispersed in the cross-linked NBR/LiTFSI (25 wt %) SPE. This result confirms that the difference in surface roughness between the front- and reverse-side surface on the SPE is mainly due to the difference in cross-linking density rather than uneven dispersion of Li^+^ ions.

**Figure 3:**
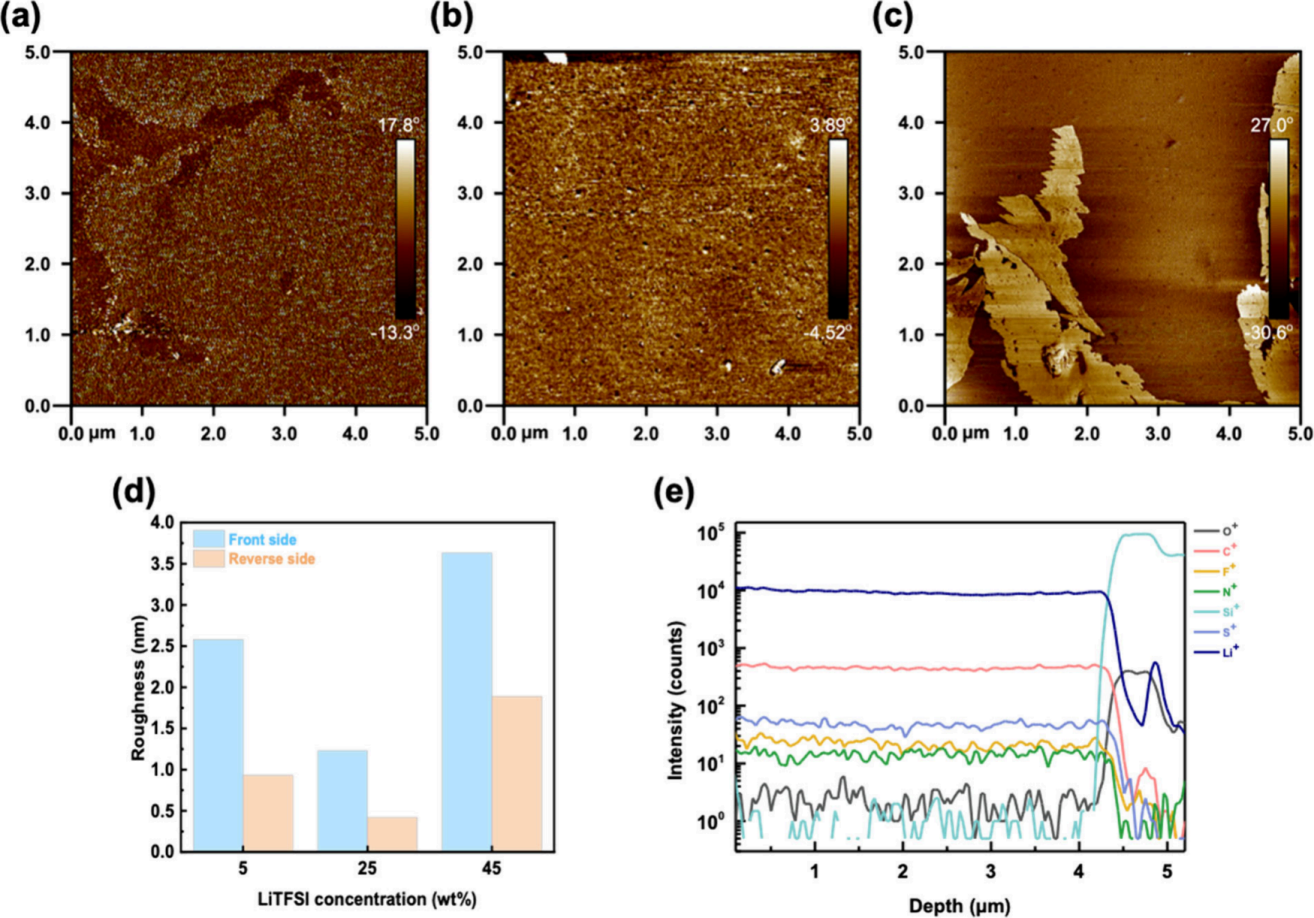
AFM phase images of the reverse-side of the cross-linked NBR-based SPE film with (a) 5 wt %, (b) 25 wt %, and (c) 45 wt % LiTFSI content. (d) Summary of root-mean-square roughness of the front and reverse sides of the VNBR-based SPE films. (e) TOF-SIMS analysis of NBR/LiTFSI (25 wt %) film.

Finally, we used the gate terminal as the presynapse, Li^+^ ions as the neurotransmitter, and the drain and source as the post-synapse to mimic the behavior of human neurons (Figure 4a). We used the gate voltage (*V*_G_ = −3 V) to analyze the SWDP behavior and investigated the synaptic responses using spike durations of 30, 60, 90, 120, and 150 ms. As shown in Figure 4b, the excitatory postsynaptic current (EPSC) increases with increasing pulse duration. The highly crystallinity of PDBT-*co*-TT made the cross-linked NBR-based SPE easier to form an electrical double layer (EDL). Then, more Li^+^ ions in the cross-linked NBR-based SPE accumulate between SPE/channel interface with increasing duration rather than diffusing into the active channel and causing irreversible effects.^46^ When the *V*_G_ is returned to the reading voltage (*V*_G_ = 0 V), the Li^+^ ions cannot immediately return to their initial state, which further produces synaptic behavior. Furthermore, we tuned the spike frequencies to 3.33, 6.25, 10, 16.7, 25, and 50 Hz, then observed a significant enhancement of the EPSC in the high-frequency region after 10 consecutive spikes, as shown in Figure 4c. We assessed the gain value by calculating the ratio of the 10th spike to the first spike at each frequency. High frequency was the most effective way to enhance the EPSC. As the frequency was increased from 3.33 to 50 Hz, the gain value increased from 0.96 to 1.91, respectively. Figure 4d exhibits that higher spike frequencies significantly enhance synaptic learning behavior. Next, we used eq 1 to describe the paired-pulse facilitation (PPF) of the synaptic transistors to evaluate the learning properties of the synapse.
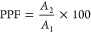
1where *A*_1_ and *A*_2_ are the magnitudes of the first and second EPSC, respectively. As presented in Figure 4e, the cross-linked NBR-based EGOFET exhibited a good learning rate of 125% at spike interval of 30 ms. The learning rate remained above 100% for intervals longer than 500 ms. The PPF decay described in eq 2 defines the synaptic forgetting rate during short-term learning:
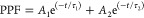
2where *t* is the pulse interval time, τ_1_ and τ_2_ are the decay times, and *A*_1_ and *A*_2_ are the facilitation magnitudes of the fast and slow phases, respectively. The results show the τ_1_ of 3.5 ms describes the fast-forgetting behavior at the beginning, and τ_2_ of 54.2 ms describes the subsequent slow-forgetting behavior. These results suggest that the cross-linked NBR-based EGOFET has synaptic plasticity and can transform the acquired information from short-term memory to long-term memory. This behavior is like the ionic conduction characteristics of biological synapses, where Li^+^ ions in the electrolyte gate electrode act as neurotransmitters. The release of Li^+^ ions is initiated by applying *V*_G_, and the release is enhanced by applying a second spike *V*_G_ before Li^+^ ions decay to equilibrium. Notably, we applied 35 consecutive writing and erasing voltage spikes to mimic the long-term synaptic properties of long-term potentiation (LTP) and long-term depression (LTD). Figure 4f shows the same long-term plasticity is also observed in terms of synaptic characteristics. Besides, we show the LTP/LTD cycling stability in Figure 4g. The EPSC increased with cycling due to Li^+^ ions diffusing in cross-linked NBR-based SPE. The cyano group enhanced ionic diffuse paths.^47,48^ The synaptic behavior of the NBR-based EGOFET is strongly influenced by the diffusion of Li^+^ ions and their interaction with the NBR matrix. The cyano groups of NBR play a crucial role in enhancing the ionic diffusion paths. This enhancement is attributed to the interaction between the Li^+^ ions and the cyano groups, which facilitates the diffusion of Li^+^ ions to the SPE/channel interface. As depicted in Figure 2a, the MEP calculations illustrate the interaction between the Li^+^ ions and the cyano groups. This interaction leads to the accumulation of Li^+^ ions at the SPE/channel interface, which, in turn, enhances the synaptic characteristics. The accumulation of Li^+^ ions at the SPE/channel interface is analogous to the accumulation of neurotransmitters in biological synapses, contributing to the synaptic plasticity of the device. This behavior is further evidenced by the observation of various synaptic behaviors such as SWDP, SRDP, PPF, and LTP/D, as shown in Figure 4. In summary, the diffusion of Li^+^ ions and their interaction with the cyano groups in the NBR matrix are essential for the synaptic behavior observed in our NBR-based EGOFETs. Then, we evaluated the energy consumption of the cross-linked NBR-based EGOFETs by eq 3.
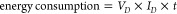
3where *V*_D_ is the driving voltage, *I*_D_ is the EPSC of each spike, and *t* is the stimulate duration. The cross-linked NBR-based EGOFETs show energy consumption as low as 15.9 nJ/spike under an electrical modulation of *V*_D_ = −1 V. This finding suggests that an artificial synapse using the cross-linked NBR-based EGOFET has excellent capabilities, in terms of memory and information processing, making it a strong candidate for future neuromorphic application.

**Figure 4:**
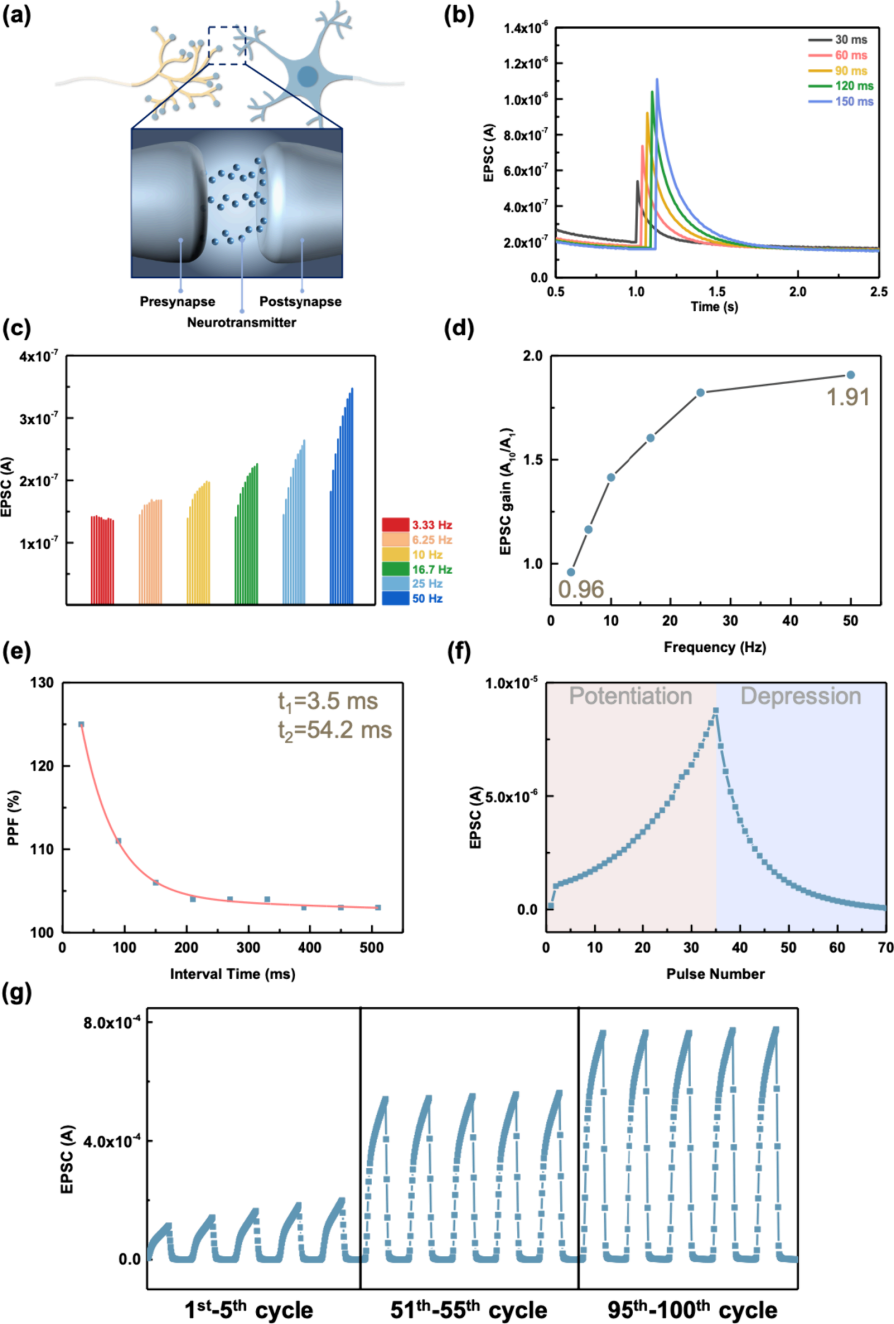
(a) Schematic diagram of the artificial synapses. Synaptic behavior of (b) spike-width dependent plasticity (SWDP), (c) spike-rate-dependent plasticity (SRDP), (d) EPSC gain at different frequencies, (e) PPF, (f) LTP/D, and (g) cyclic stability of the cross-linked NBR-based EGOFETs.

Furthermore, we demonstrated the potential of the cross-linked NBR-based EGOFET for practical neuromorphic application by constructing a deep neural network (DNN) based on handwritten digit recognition from the Modified National Institute of Standards and Technology (MNIST). The configuration of the DNN is shown in the illustration of Figure 5a. As shown in Figure 5b, the numbers displayed in each position represent the actual classified numbers; the columns correspond to the identification categories classified by the DNN, and the rows correspond to the categories of the test data. Most of the handwritten digits were correctly classified as the corresponding digits (0–9). Figure 5c shows that the DNN weights based on the cross-linked NBR-based EGOFET achieved a recognition rate of 91.9% after 50 training epochs. Figure 5d shows the reliability of DNN, which further converges with increasing training times and has minimal overfitting characteristics. We further validated the DNN’s ability to recognize unfamiliar handwritten digit images. Figure 5e shows the cognitive results of DNN based on the cross-linked NBR-based EGOFETs. The results show that the device can perform complex recognition tasks in neuromorphic computing. Furthermore, a high recognition rate (91.9%) can be achieved in a relatively short training period. We have compared *I*_on_/*I*_off_, recognition, and energy consumption with other reports of SPE-based EGOFET (Table S2). Overall, our results show that the cross-linked NBR-based EGOFETs are a promising approach for developing efficient and powerful neuromorphic devices.

**Figure 5:**
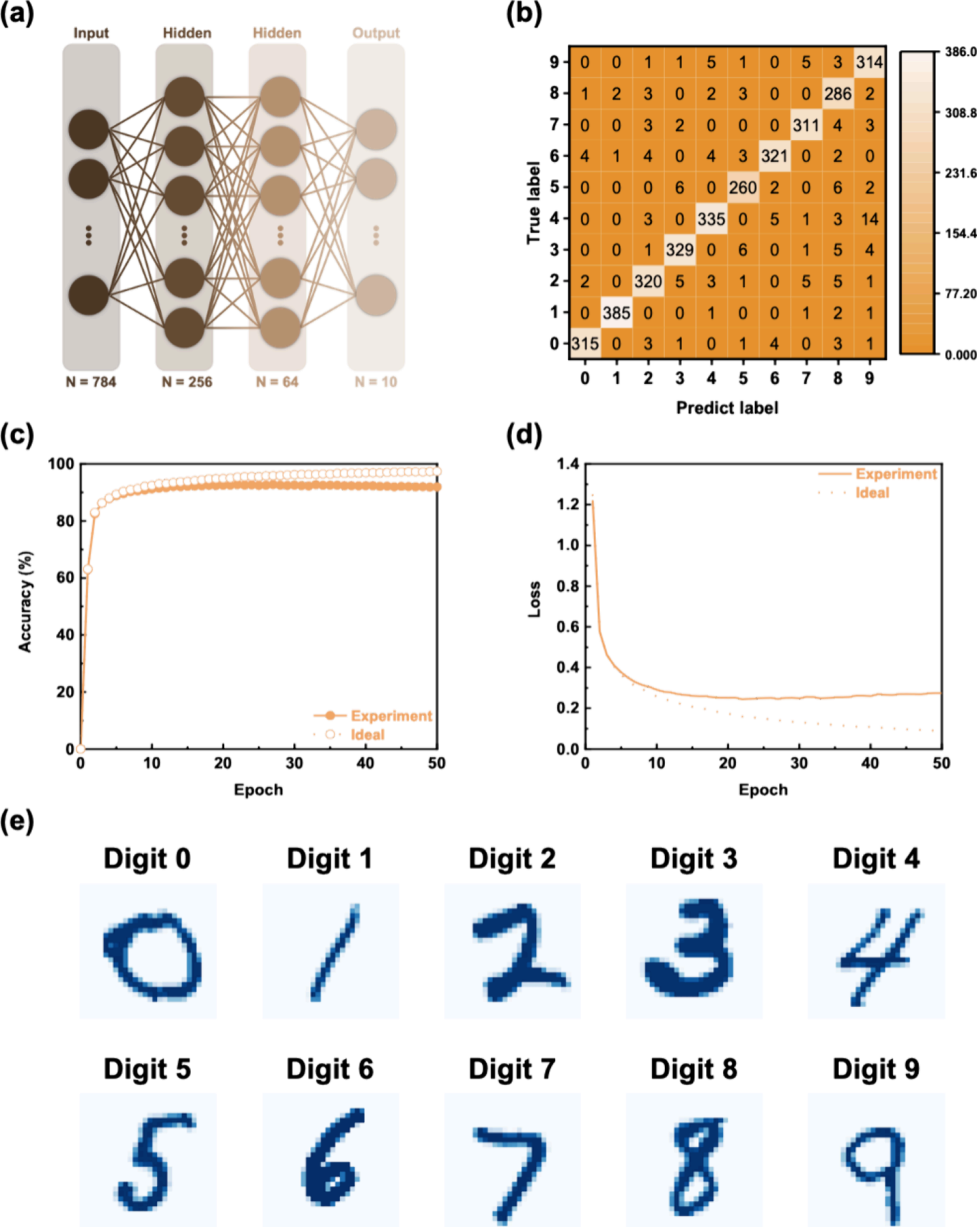
(a) Schematic of the DNN architecture. (b) Confusion matrix for handwritten digit classification after training 50 epochs. (c) Recognition accuracy, and (d) loss plots of the cross-linked NBR-based EGOFET during training. (e) DNN-based handwritten digit classification results after training.

In summary, we successfully used photoinduced thiol–ene radical reactions to prepare a novel SPE for the preparation of a cross-linked NBR network, which was doped with LiTFSI for EGOFETs. The NBR-based EGOFETs exhibited high transconductance (*g*_m_ = 11.9 mS) and high on/off current ratio (*I*_on_/*I*_off_ = 10^5^), which was achieved by controlling the surface roughness (0.42 nm) and scan rate (40 mV/s). In addition, the EGOFET based on the cross-linked NBR/LiTFSI electrolyte film successfully demonstrated various artificial synaptic behaviors, including SWDP, SRDP, PPF and LTP/D, with low-energy consumption of 15.9 nJ/spike. Finally, we demonstrated the potential of DNN based on the cross-linked NBR devices for handwritten digit recognition. After 50 training cycles, the model achieved a recognition rate of 91.9%, and its ability to classify unfamiliar data was demonstrated by the confusion matrix. This work shows that our proposed photopatternable NBR-based SPE presents a promising approach for developing large-scale, low-energy neuromorphic devices.

## Supporting Information Available

All materials and EGOFET fabrication and characterizations; schematic diagram of EGOFET; additional capacitive response, impedance spectra, transfer curves, output curves, AFM images; comparative performance of synaptic devices (PDF)
