# Supplementary material for: Organic Solid-State Electrolyte Synaptic Transistors with Photoinduced Thiol–Ene Cross-linked Polymer Electrolytes for Deep Neural Networks
Source: ACS Mater Lett. 2025 Jan 23;7(2):682–91. doi: 10.1021/acsmaterialslett.4c02511 (PMC11795873; doi:10.1021/acsmaterialslett.4c02511)
Supplement: Supplementary file 1 — tz4c02511_si_001.pdf [file tz4c02511_si_001.pdf]

Supporting Information

Organic Solid-State Electrolyte Synaptic Transistors with Photoinduced Thiol-Ene Crosslinked Polymer Electrolytes for Deep Neural Networks

Qun-Gao Chen,[a,†] *Wei-Ting Liao,*[a,†] *Rou-Yi Li,*[a] *Ignacio Sanjuán,*[b] *Ning-Cian Hsiao,*[a] *Chan-Tat Ng,*[c] *Ting-Ting Chang,*[c,d] *Antonio Guerrero,*[b] *Chu-Chen Chueh,*[e,*] *Wen-Ya Lee*[a,d*]

[a] Department of Chemical Engineering and Biotechnology, National Taipei University of Technology, Taipei 106344, Taiwan

[b] Institute of Advanced Materials (INAM), Universitat Jaume I, 12006 Castelló, Spain

[c] Department of Psychology, National Chengchi University, Taipei, Taiwan

[d] Research Center for Mind, Brain & Learning, National Chengchi University, Taipei, Taiwan

[e] Department of Chemical Engineering, National Taiwan University, Taipei 10617, Taiwan

Keywords: electrolyte-gated organic field-effect transistor; electrochemical double layers; solid-state electrolyte; artificial synapse; neuromorphic computing

Experiment Section

Materials

The processing solvent, such as chlorobenzene (CB), n-Butyl acetate (BAC) were purchased from Sigma-Aldrich and used as received. Acetonitrile (ACN) was purchased from Honeywell and used as received. The cleaning solvent, such as toluene, acetone, and isopropanol (IPA) were purchased from Mallinckrodt. Poly(acrylonitrile-*co*-butadiene) (NBR) with acrylonitrile 37-39 wt.%, poly(vinylidene fluoride-*co*-hexafluoropropylene) (PVDF-HFP), poly(ethylene oxide) (PEO), lithium bis(trifluoromethanesulfonyl)imide (LiTFSI), diphenyl(2,4,6-trimethylbenzoyl)phosphine oxide (TPO), trimethylolpropane tris(3-mercaptopropionate) (TMPMP), and poly(3,4-ethylenedioxythiophene) polystyrene sulfonate (PEDOT:PSS) were purchased from Sigma-Aldrich and used as received. N-octadecyltrimethoxysilane (OTMS) was purchased from Gelest Inc. Poly(2,2'-[(2,5-bis(2-octyldoecyl)-3,6-dioxo-2,3,5,6-tetrahydropyrrolo[3,4-c]pyrrole-1,4-diyl)]dithiophene-5,5'-diyl-*alt*-thieno[3,2-b]thiophene-2,5-diyl) (PDBT-*co*-TT) was purchased from Luminescence technology corp.

EGOFET fabrication and characterizations

The electrolyte-gated organic field-effect transistors (EGOFETs) have a top-gate/top-contact construction. We formulated the polymer electrolyte solution using the following materials:

NBR, PVDF-HFP, and PEO were doped with LiTFSI (5/ 25/ 45 wt.%) as the electrolyte, respectively. Then, adding TPO as the photoinitiator, and TMPMP as the photo-crosslinking agent. Subsequently, the SiO_2/Si wafers threated with OTMS were then cut into 0.6 × 0.8 cm^2 pieces. The appropriate volume of dielectric layer solution was then carefully applied directly to the wafer surface. The wafers were then left at room temperature for at least 8 hours. At the end of the waiting period, the NBR/LiTFSI SPE was exposed to UV curing for 5 minutes to initiate the crosslinking reaction. Subsequently, the film was gently peeled off and affixed to the pre-treated semiconductor layer alongside the coated electrodes. Likewise, PVDF-HFP and PEO films with LiTFSI were peeled off and attached to the prepared semiconductor layer and coated electrodes after standing for more than 8 hours. Finally, the entire device was completed by dropping PEDOT:PSS onto the SPE as the gate electrode. All the transistor properties were measured in atmosphere using a Keithley 2634B.

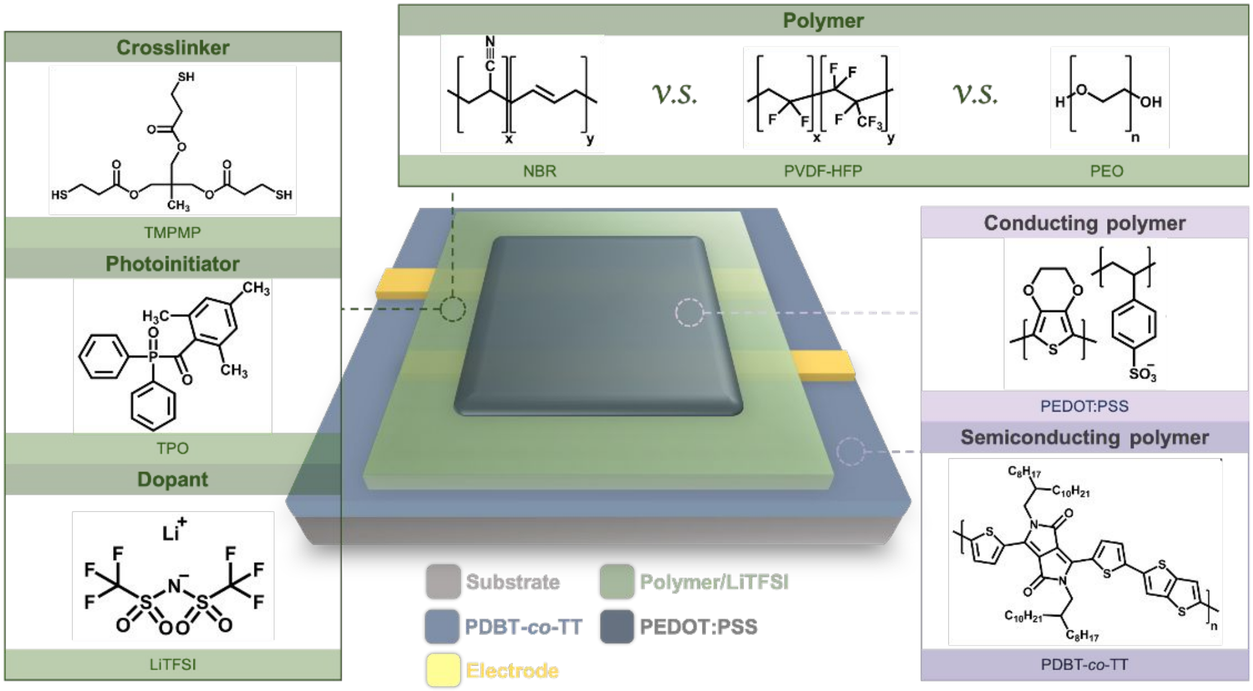

Figure S1. Schematic of EGOFET structure; chemical structures of the solid-state polymer electrolytes, conjugated polymer, and conducting polymer.

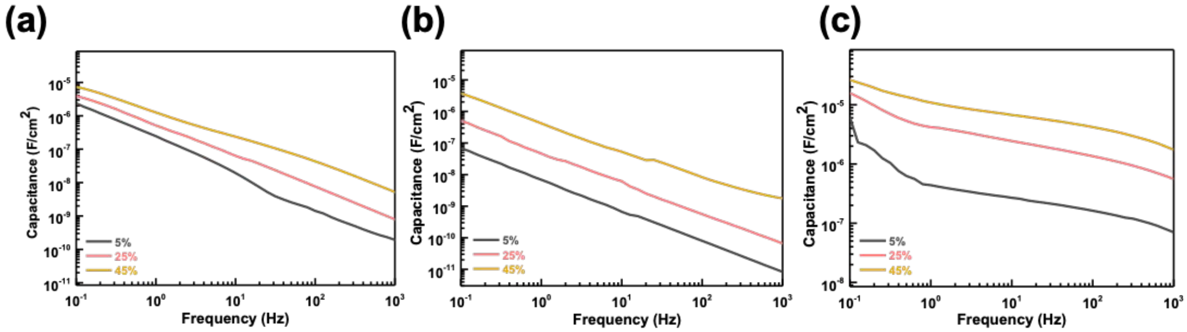

Figure S2. Capacitive response of LiTFSI concentration on elastomers based on (a) crosslinked NBR, (b) PVDF-HFP, and (c) PEO.

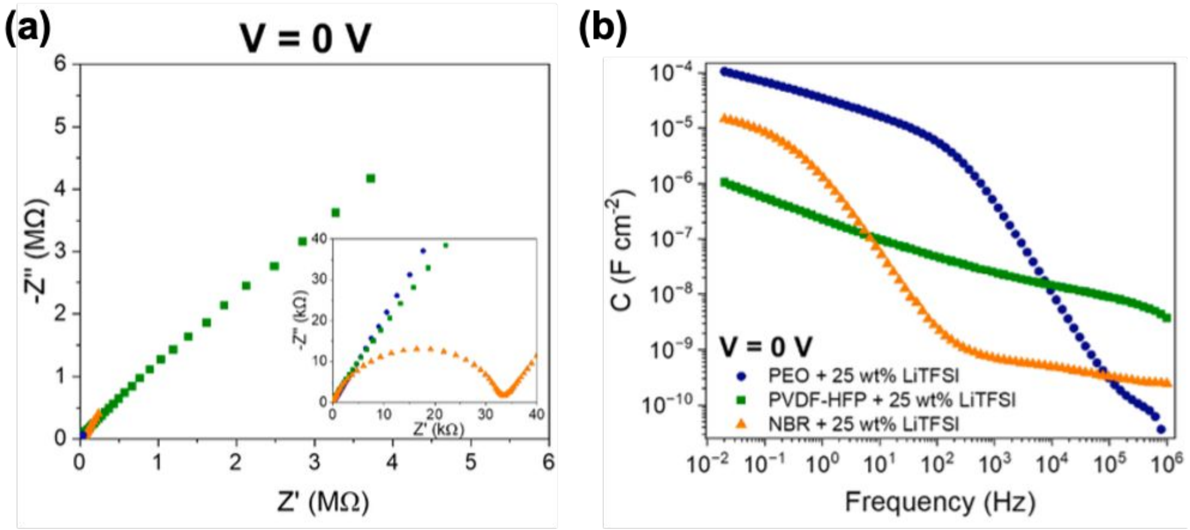

Figure S3. (a) Complex plane plot representation of the impedance spectra and (b) plot of capacitance versus frequency for the three polymers at an applied DC voltage of 0 V.

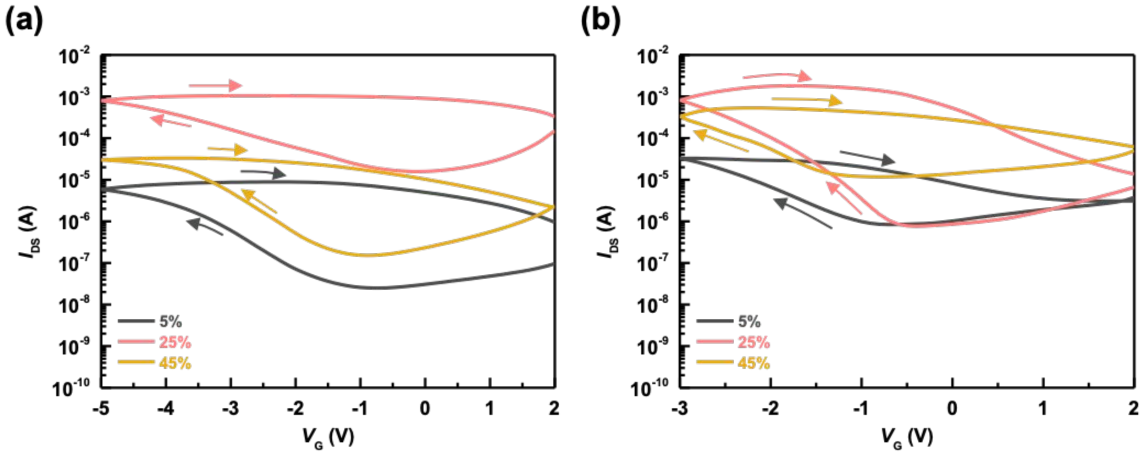

Figure S4. Transfer curves of EGOFETs based on (a) PVDF-HFP, and (b) PEO with different LiTFSI contents (5, 25, 45 wt.%) films.

Table S1. Ionic conductivities of the polymer/LiTFSI-based electrolytes with 25 wt.% LiTFSI.

**Polymer**	**Resistivity (Ω × cm)**	**Conductivity[a] (S/cm)**
Crosslinked NBR	5.14 ×106	1.95 ×10-7
PVDF-HFP	8.21 ×106	1.22 ×10-7
PEO	3.22 ×104	3.11 ×10-5

[a] The ionic conductivities are calculated through Pouillet's law, and the area of SPEs used for EIS measurements is 1.77 cm2.

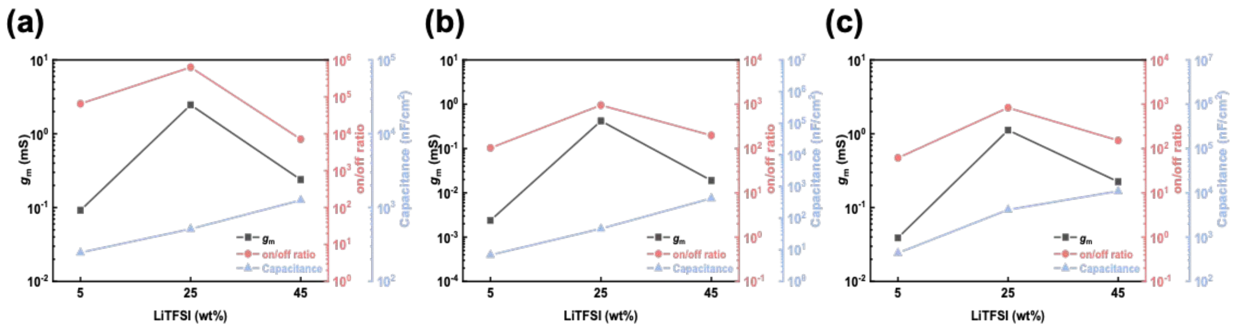

Figure S5. Comparison of device performance of PDBT-*co*-TT EGOFETs based on (a) crosslinked NBR, (b) PVDF-HFP/LiTFSI and (c) PEO/LiTFSI electrolytes with different LiTFSI doping concentrations.

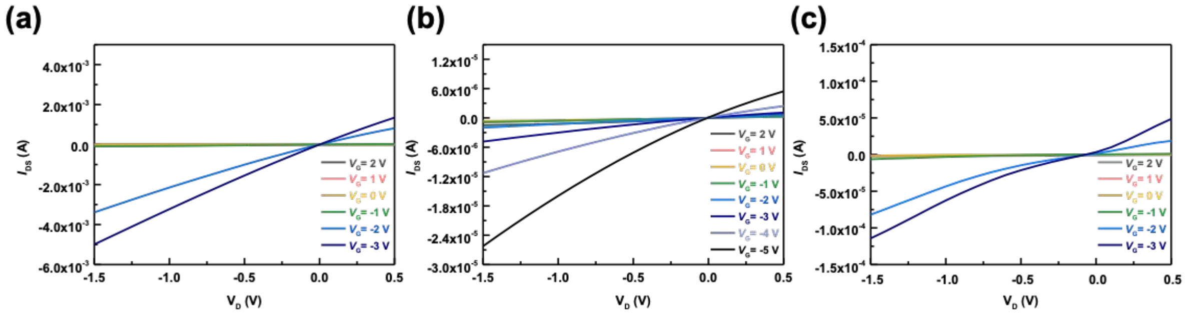

Figure S6. Output characteristics of PDBT-*co*-TT EGOFETs based on (a) crosslinked NBR, (b) PVDF-HFP, and (c) PEO electrolytes with 25 wt.% LiTFSI.

(a)

(b)

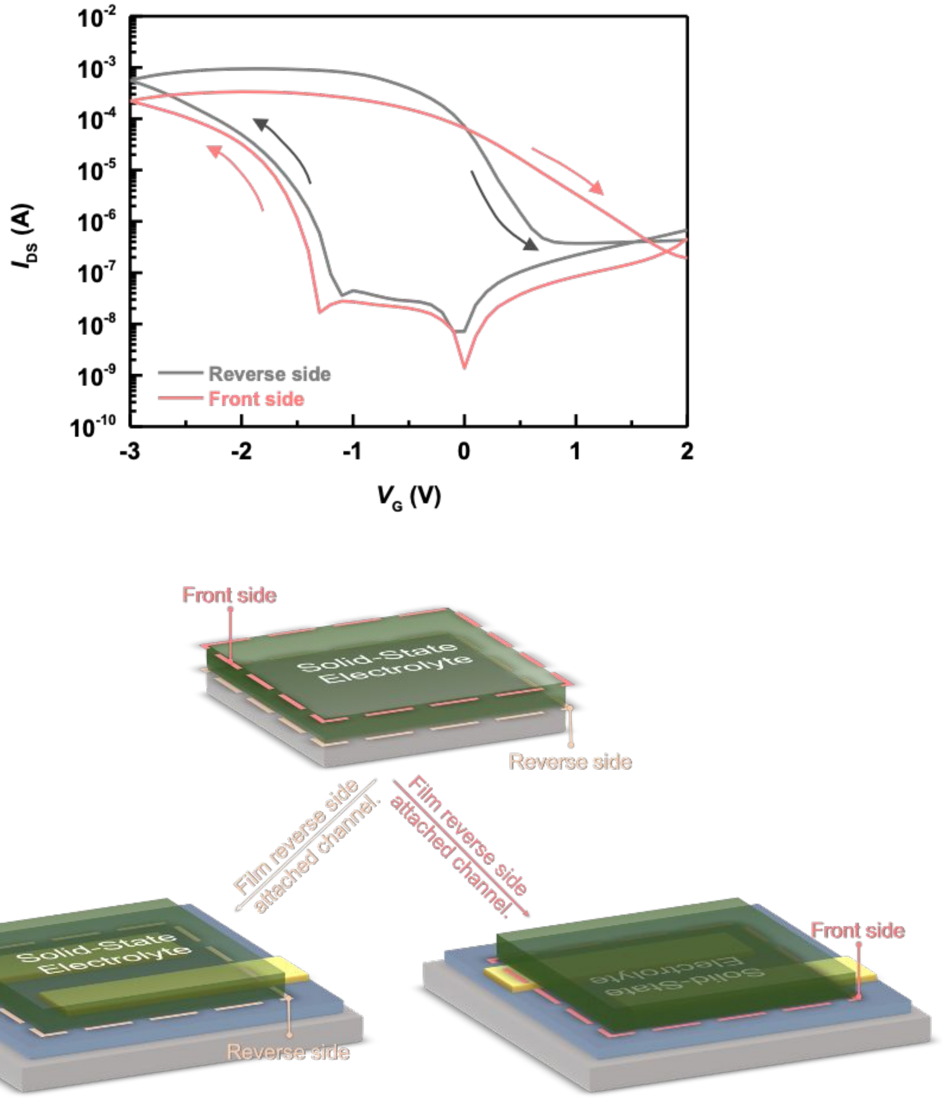

Figure S7. (a) Transfer curves of PDBT-*co*-TT EGOFET based on the crosslinked NBR/LiTFSI (25 wt.%) electrolyte film with either the front or the reverse side in contact with the active channel. (b) Schematic of the front and reverse sides of the crosslinked NBR/LiTFSI electrolyte film.

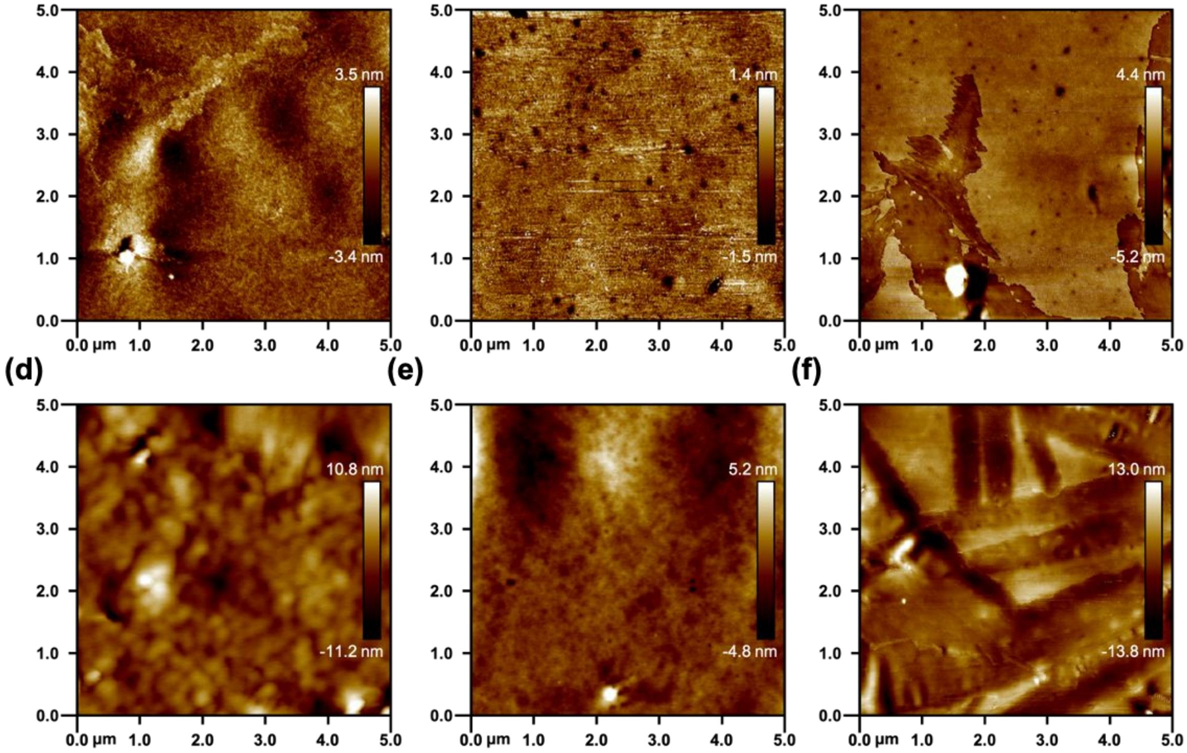

Figure S8. AFM height images of (a-c) the reverse side and (d-f) the front side of the crosslinked NBR/LiTFSI electrolyte films with (a,d) 5 wt.%, (b,e) 25 wt.%, and (c,f) 45 wt.% LiTFSI doping content.

Table S2. Literature comparison of EGOFETs.

Ref.	Polymer	Ions	I_{on}/I_{off}	Accuracy (%)	Energy consumption
1	PVDF-HFP	$[BMIM]^-[TFSI]^-$	10^4	N/A	N/A
2	PVDF-TrFE	$[Li]^+[TFSI]^-$	10^5	89.13	N/A
3	PVA	$[Li]^+[TFSI]^-$	10^6	75.96	7.29 pJ/spike
4	PVA	Lignin	10^6	N/A	0.63 nJ/spike
5	PEO	$[IA]^+[Perchlorates]^-$	10^3	N/A	N/A
6	PAN	$[Li]^+[TFSI]^-$	10^3	N/A	27.2 nJ/spike
**This work**	NBR	$[Li]^+[TFSI]$	10^5~10^6	91.9	15.9 nJ/spike

References

1. L. Lan, J. Chen, Y. Wang, P. Li, Y. Yu, G. Zhu, Z. Li, T. Lei, W. Yue and I. McCulloch, *Chem. Mater.*, 2022, 34, 4, 1666-1676.

2. M. Jin, H. Lee, J. H. Lee, D. Han, C. Im, J. Kim, M. Jeon, E. Lee and Y. S. Kim, *Appl. Phys. Rev.*, 2023, 10, 011407.

3. J. Huang, J. Chen, R. Yu, Y. Zhou, Q. Yang, E. Li, Q. Chen, H. Chen and T. Guo, *Org. Electron.*, 2021, 89, 106019.

4. W. Zhang, J. Li, L. Cheng, W. Shi, Y. Lei, S. Wen, F. Wang, J. Jiang, P. Wen and J. Zhang, *IEEE Trans. Electron Devices*, 2023, 70, 6, 3245-3250.

5. J. Sun, Y. Deng, J. Jiang, Y. Hu, Q. Cui, Z. Lou, Y. Hou, F. Teng, *Org. Electron.*, 2022, 106, 106529.

6. X. Wang, Y. Yan, E. Li, Y. Liu, D. Lai, Z. Lin, Y. Liu, H. Chen and T. Guo, *Nano Energy*, 2020, 75, 104952.
